# Early-Occurring Dendritic Spines Alterations in Mouse Models of Alzheimer’s Disease Inform on Primary Causes of Neurodegeneration

**DOI:** 10.3389/fnsyn.2020.566615

**Published:** 2020-09-10

**Authors:** Martine Ammassari-Teule

**Affiliations:** ^1^Institute of Biochemistry and Cell Biology, CNR-National Research Council, Rome, Italy; ^2^Laboratory of Psychobiology, IRCCS Santa Lucia Foundation, Rome, Italy

**Keywords:** Alzheimer’s disease, presymptomatic stage, full-length APP, Aβ oligomers (Aβo), dendritic spines, synaptic proteins

## Abstract

The consensus that synaptic failure is the earliest cause of cognitive deterioration in Alzheimer’s disease (AD) has initially led to investigate structural (dendritic spines) and physiological (LTP) synaptic dysfunctions in mouse models of AD with established cognitive alterations. The challenge is now to track down ultra-early alterations in spines to uncover causes rather than disease’s symptoms. This review article pinpoints dysregulations of the postsynaptic density (PSD) protein network which alter the morphology and function of spines in pre- and early- symptomatic hAPP mouse models of AD, and, hence, inform on primary causes of neurodegeneration.

## Introduction

Re-arrangements of synaptic connectivity in neural circuits of memory allow individual experiences to be encoded, stored, and subsequently recalled. These re-arrangements occur at the level of dendritic spines (spines), the neuron’s dendrites protrusions which host excitatory synapses and can change their number, volume, and shape in response to environmental stimuli. The morphological plasticity of spines is supported by the dynamic properties of their actin cytoskeleton. Close to the presynaptic active zone, the postsynaptic density (PSD) of spines contains receptors, cytoskeletal, scaffolding/adaptors proteins, and signaling molecules representative of multiple pathways which control synaptic activity by maintaining a physiological balance between the morphology and the function of spines (Lee et al., 2012). Changes in shape, therefore, affect the electrical properties of spines (Tønnesen and Nägerl, 2016), modify the amount of excitatory neurotransmission (Yuste et al., 2000), and alter the wiring diagram of memory circuits through a redistribution of synaptic weights in neuronal ensembles (Varga et al., 2011; Rochefort and Konnerth, 2012).

In Alzheimer’s disease (AD), data showing that episodic memory is altered in the absence of massive neurodegeneration have established that synaptic failure is the earliest cause of cognitive deterioration (Selkoe, 2002). Hence, the search for alterations in the PSD protein network involved in structural (spines) and physiological (LTP) synaptic dysfunctions has become mainstream in the AD field (Tackenberg et al., 2009; Yu and Lu, 2012). Being mostly accessible in mouse models of the disease, these alterations have been investigated in mice overexpressing mutant human genes—amyloid protein precursor (APP), Presenilin-1 (PS1), and microtubule-associated protein Tau known to cause clinical and neuropathological alterations in familiar forms of AD. The production of mutant mice bearing single or several transgenes that were expressed in distinct wild-type backgrounds has, however, generated a variety of phenotypes that differ in the onset, the progression and the severity of AD-like symptoms (Hsiao et al., 1996). Thus, a myriad of factors impacting on structural and functional synaptic plasticity have been identified in AD mice (Tu et al., 2014; Aoki and Sherpa, 2017) and patients (Gong and Lippa, 2010), but at different ages and in association with variable neurodegenerative burdens.

Although these investigations have put forward the disruptive role of a variety molecular and biological components that alter the functionality of spines—and are suitable to be targeted in a therapeutic perspective-, the current challenge is to uncover the mechanisms that initiate the earliest spine dysfunctions with the double objective of: (i) unveiling causes rather than disease’s symptoms; and (ii) intervening to prevent rather than to contrast neurodegeneration (Llorens-Martin et al., 2014). Considering that the presence of circulating Aβ oligomers (Aβo) associates with the early signs synapto-toxicity (Lambert et al., 1998; Hsia et al., 1999; Mucke et al., 2000; Beckman et al., 2019), this review pinpoints dysregulations of the PSD protein network which disrupt the morphology and function of spines in early developing *in vitro* and *in vivo* human mutant APP models, and can inform on primary causes of neurodegeneration.

## Aβ-Induced Alteration of the PSD Network Disrupts Dendritic Spines in Cultured Neurons

### Wild-Type Neurons Exposed to Aβo

Aβo selectively bind the postsynaptic component of excitatory synapses (Lacor et al., 2007; Renner et al., 2010; Cline et al., 2018), and exposure of wild-type neurons in culture to Aβo is sufficient to trigger *in vitro* “phenocopies” of AD spines pathology. For example, pyramidal neurons in rat hippocampal organotypic slices exposed to picomolar levels (~100–300 pM) of soluble Aβo show a decrease in spine density and in the number of electrophysiologically active synapses in association with a reduction of NMDA receptor-mediated calcium influx. Of note, these effects are reverted by Aβ-specific antibodies, or by the small molecule scylloinositol (ASD 103) which prevents Aβ aggregation (Shankar et al., 2007). More recently, acute exposure to Aβo (100 nM) was found to increase phosphorylation of the cofilin-1 protein in the postsynaptic compartment and to favor its distribution in the Triton-insoluble fractions, with consequent stabilization of *F*-actin in spines, impairment of synaptic plasticity, and disruption of synaptic transmission due to the formation of cofilin-actin rods (Rush et al., 2018). Also, insertion of Aβ derived diffusible ligands (ADDL, 500 nM) in the medium of cultured mature cortical pyramidal neurons induces spine dysgenesis associated with depletion of kalirin-7, a key regulator of spines formation while overexpression of kalirin-7 prevents ADDL-induced spine degeneration (Xie et al., 2019). Data showing that synthetic and human-derived Aβo stimulates microglia proliferation at subneurotoxic nanomolar (250 nM) concentrations (Neniskyte et al., 2011) in wild-type neurons suggest that neuroinflammation plays an early role in Aβo-related neuronal subcellular alterations. Consistent with this view, hippocampal neurons in cultures exposed to Aβo in a microglia-conditioned medium show reduced levels of dendritic proteins Ac-TN and MAP2, postsynaptic proteins PSD95 and GRIP1, and presynaptic protein synaptophysin (Maezawa et al., 2011). In apparent contrast with these findings, *in vivo* time-lapse imaging of spines in organotypic hippocampal slices treated with micromolar (1 μM) concentrations of Aβo reveals an increase in the density of total dendritic spines due, however, to a rise of immature stubby spines with no head and neck differentiation preventing synaptic signal compartmentalization and therefore unlikely to enhance synaptic transmission (Ortiz-Sanz et al., 2020). Suggesting that the toxicity of Aβo is independent of its subcellular localization, overproduction of axonal or dendritic Aβo in rat cultured cortical neurons disrupts both synaptic density and plasticity (Wei et al., 2010). Aβo (10 μM) also impact scaffolding and cytoskeletal proteins in cortical neurons in culture. Specifically, a reduction in PSD-95 levels at glutamatergic synapses (Roselli et al., 2005), a disassembly of Homer1b and Shank1, two scaffold proteins that couple PSD-95 with ionotropic and metabotropic glutamate receptors (Roselli et al., 2009), and a significant reduction in the density and morphology of spines accompanied by decreased levels of the spine cytoskeletal protein drebrin (Lacor et al., 2007) have been reported.

### Mutant hAPP Neurons

Compared to wild-type neurons exposed to Aβo, neurons from AD mice have the advantage to inform on the chronology of synaptic alterations whose severity varies according to: (i) the age and the neurodegenerative burden of the mouse model used for the preparation of neurons in culture; and (ii) the degree of maturation of cells, which depends on the number of days they are kept growing *in vitro* (DIV). For example, in cultured neurons from Tg2576 cortices and hippocampi prepared at embryonic days 16 and 18, the first synaptic alterations are detected on the postsynaptic side. Those consist in a synaptic reduction of PSD-95 and glutamate receptor subunit GluA1 levels observed at 12 DIV, which become more severe at 19 DIV since not only synaptic but total levels of PSD-95 and GluR1 are decreased and fewer spines, identified by PSD-95, spinophilin, drebrin, and *F*-actin staining are counted. Interestingly, 19 DIV is also the time point where the first presynaptic alteration, i.e., decreased levels of synaptophysin, is detected (Almeida et al., 2005). To better intercept *in vitro* developmental changes in spines, Penazzi et al. (2016) established a long term *ex vivo* model of hippocampal slices prepared from 7- or 14-day-old mice that were kept in culture for 15 or 20 days. Spine loss and progressive changes from mushroom- to stubby- shaped spines in CA1 hippocampal neurons were observed in all preparations, but these dysfunctions were considerably stronger in the P14/20 DIV condition. Of note, the changes in spines observed in cultured neurons were fully consistent with those exhibited by the same mice at 3- 4- and 5-week of age. Confirming that the most severe alterations occur in the most mature AD neurons, primary cortical neurons prepared from postnatal day 1 APP/PS1 mice and fixed at DIV 10 and 16 revealed that while only the spine head diameters were reduced at DIV 10, head diameters, surface areas, and *F*-actin levels were reduced in spines measured at DIV16 (Kommaddi et al., 2018). Then, consistent with data showing that spine loss in AD patients and mice models occur through a pathway involving the calcium-dependent phosphatase calcineurin (CaN), neurons isolated in the vicinity of plaques and further exposed to soluble Aβo was found to activate CaN, which then activated the transcriptional factor nuclear factor of activated T cells (NFAT) involved in Ca^(2+)^ dysregulation (Abdul et al., 2009). Remarkably, even in the absence of Aβo, the activation of CaN or NFAT pathways is sufficient to produce dystrophic neurites, dendritic simplification, and dendritic spine loss (Wu et al., 2010).

## Aβ-Induced Early Disruption of PSD Network and Spines in Resting Conditions

### Asymptomatic Stage

The earliest evidence of spine loss in a mouse model of AD is provided by Lanz et al. (2003) who reported a decrease in spines in Golgi-stained pyramidal neurons in the CA1 subfield of the hippocampus in 2-month-old PDAPP mice expressing the V717F hAPP mutation in a C57BL/6xDBA/2xSwiss-Webster background. Of note, the loss of spines was detected only at the age of 3 months in Tg2576 mice expressing the same mutation but in a C57BL6xSJL6 background. More recently, in a study aimed at investigating the time course of entorhinal cortex (EC) dysfunction in AD mice, we measured synaptic activity/plasticity and dendritic spines in 2- and 6-month-old hAPP bearing the FAD Swedish and Indiana APP mutations (Criscuolo et al., 2017). Field potentials recorded in EC superficial Layer II after stimulation of the same layer revealed that input-output curves and LTD were unaltered while LTP was absent in *2-month-old* mutants. Consistent with the LTP deficit, a massive increase in the number of immature thin spines lacking post-synaptic components was observed in the same layer. Interestingly, these synaptic defects were associated with a selective impairment in the EC-dependent novel object-place-context recognition task. At the age of 6 months, all electrophysiological parameters were altered and mice were impaired in all recognition memory tasks, including the simple reactivity to a novel object introduced at the same place and context of a previously explored object). By showing that the LTP deficit precedes impairments in basal transmission and LTD, but associates with spine loss, these *in vivo* observations confirm the aforementioned *in vitro* data (Almeida et al., 2005) that AD synaptic failure initiates by dysregulation of postsynaptic elements. Indeed, abnormalities in PSD cytoskeletal proteins contribute to early spine loss. For example, at the same age, hAPP mice show an enhancement of phosphorylated p38 MAPKinase, a key regulator of pro-inflammatory cytokines involved in multiple aspects of cell physiology including cytoskeleton remodeling (Cuenda and Rousseau, 2007). Even earlier, Kommaddi et al. (2018) report depolymerization of *F*-actin accompanied by increased globular-actin (G-actin) in *1-month-old* APPswe/PS1ΔE9 mice. Confirming the primary role of cytoskeletal, alterations in synaptic deterioration, the levels of the scaffold proteins PSD95 and homer1, and of the glutamate receptor subunit GluA1 are unaltered. Supporting the functional role of *F*-actin in spines, the impairment in contextual fear conditioning (CFC) recall shown by APP/PS1 mice at *2 months* of age was rescued by *in situ* injections of the actin-polymerizing agent jasplakinolide. Although no visualization and measurement of spines were carried out in this model, direct stochastic optical reconstruction microscopy (dSTORM) revealed that *F*-actin depolymerization strongly disrupts the nano-organization of radiating *F*-actin rods in spines imaged on cortical neuron dendrites. The disruptive effect of actin disassembly on the morphology and function of cortical synapses in 2-month-old mutants led the authors to conclude that *F*-actin depolymerization is causal and not consequential to spine disruption (Kommaddi et al., 2018).

### Early Symptomatic Stage

Around 3 months of age, all mice bearing hAPP mutations alone or in association with PS1 and Tau mutations show elevated levels of soluble Aβo which significantly impact the structural and plastic properties of spines in key brain regions for cognition. Disruption of hippocampal circuits in 3-month-old APP_ind_ mutants due to synaptic protein alterations was first reported by Hsia et al. (1999). Although dendritic spines were not measured in this study, a deficit in synaptic transmission was associated with a reduction of synaptophysin in presynaptic terminals and in the number of microtubule-associated protein two-positive neurons in the CA1 region revealed that dysfunctions in the PSD protein network were already present in these young mutants. Remarkably, the addition of the Swedish transgene to the Indiana mutation increased synaptic transmission deficits. More recently, evidence has accumulated that 3-month-old hAPP mutants show a reduction in hippocampal and/or cortical spines in association with abnormal caspase-3 accumulation (D’Amelio et al., 2011), *F*-actin disassembly (Kommaddi et al., 2018), or upregulation of full-length APP translation (Borreca et al., 2020). Further support to the link between Aβ-induced alterations in the PSD network and disruption of synaptic plasticity comes from data which show that phosphorylation of the actin-binding protein cofilin-1 (pcof1) is increased in the postsynaptic enriched fraction of cortical synaptosomes of 3-month-old APP/PS1 mice. Complementary immunohistochemical investigations revealed that p-cof1 is increased in cortical spines which otherwise showed larger synaptic areas. The authors conclude that, as in cortical neurons in culture, Aβo-induced excessive stabilization of actin in spines which prevents their plastic remodeling in response to stimuli (Rush et al., 2018). At the same age, mice homozygous for the PS2APP transgene show an abnormal contribution of GluN2B NMDA receptors to hippocampal synaptic plasticity compared to the wild-type mice (Hanson et al., 2015) while dynamics imaging of spines in the cerebral cortex of APPswe/PS1ΔE9 mice reveals an aberrant enhancement of spine turnover associated with a decrease in the number of persistent spines (Heiss et al., 2017). In the same mice, a reduction in dendritic calcium activity and in the size of spines is detected in the primary motor cortex during quiet resting but not during treadmill running which suggests some form of activity-dependent recovery specific to motor behavior (Bai et al., 2017). Then, consistent with the view that morphological alterations of spines trigger synaptic failure, Androuin et al. (2018) report that spines measured in 3-month-old APP/PS1 knock-in mice exhibit a reduction in length and an enlargement of necks before the diminution of synaptic density in the stratum radiatum layer of the hippocampus. Mathematical modeling of these data suggests that these morphological changes disrupt the electrical compartmentalization of spines and produce a selective diminution of postsynaptic potentials in spine heads required for LTP. This hypothesis is then confirmed experimentally by showing that LTP, but not basal transmission, is impaired in hippocampal slices from these mutant mice.

## Cognitive Activity Discloses Or Anticipates Observation of Aβ-Induced Spine Disruption

The paradox of studies aimed at investigating structural synaptic plasticity defects in AD mice is that despite data that show that activity-triggered plasticity relates directly to cognitive processing (Kasai et al., 2010), spines in AD mouse models have scarcely been examined under cognitive challenge. If memory requires changes in neuronal networks based on modifications in strength and number of synapses, better characterization of synaptic defects underlying memory dysfunction in AD mice are expected to emerge during or immediately after mice are asked to form memories. Because the state of activation of synapses affects Aβ homeostasis (Cirrito et al., 2005; Tampellini, 2015) which, in turn, increases Aβo secretion and depresses excitatory synaptic transmission, it is of primary importance determining which age cognitive activity-induced release of Aβo starts to impact synapses in hAPP mutants, and how brain circuits reorganize to contrast early synaptic dysfunctions.

### APP 23 Mice Show Regular Hippocampal Spines at Rest but Form More Training-Induced Spines

Middei et al. (2010) provided the first evidence that training produces compensatory reshaping of memory circuits in a mouse model of AD. Seven-month-old APP23 mice trained in a water maze were found to travel a longer distance to find the submerged platform compared to wild-type mice. After the experiments, mice were sacrificed to evaluate the impact of spatial training on dendritic spines and synaptic plasticity in the hippocampus. Spine density did not differ between mutant mice and wild-type mice in the control cage and pseudo-training conditions. In the training condition, all mice showed a posttraining increase in spines which indicates that reactive plasticity was spared by the mutation. The increase in spines was, however, stronger in the mutant mice than in the wild-type mice. Synaptic plasticity measured in slices from pseudo-trained mice revealed no difference in LTP induction and maintenance between genotypes. In slices from trained mice, LTP was regularly induced in both genotypes but decayed more rapidly in the mutant mice. These findings reveal that hAPP circuits are unaltered in non-training conditions but undergo compensatory stronger remodeling in training conditions to sustain a less efficient, likely more effortful, cognitive performance. Thus, if more spines are formed in trained mutant mice to compensate for the disruptive effect of training-induced Aβo release at hippocampal synapses, it could be the decay in LTP maintenance depends on the training mobilization of more synapses which reduces the pool of synapses available for LTP.

### Cognitive Activity Enhances Aβ Release in the Hippocampus of 2-Month-Old Tg2576 Mice and Prevents the Learning-Induced Formation of Spines

We previously reported that 2-month-old Tg2576 show intact density and morphology of hippocampal spines at rest, and regular CFC when returned 1 day after to the safety training context (D’Amelio et al., 2011). Measurements of spines in the CFC circuitry upon CFC recall reveal however that, differently from wild-type mice, Tg2576 mice do not show any increase in mushroom spines in the CA1 region of the hippocampus. Conversely, both wild-type and Tg2576 mice show an increase in mushroom spines in the BLA region of the amygdala, but the presence of an additional increase in BLA thin spines in Tg2576 mice indicates more substantial BLA rewiring in the mutant mice. Dot blot quantification of Aβlevels in each region 24 h after CFC encoding reveals that lack of CFC-induced hippocampal spines in the mutant mice can be ascribed to the selective vulnerability of their hippocampus to the activity-induced release of Aβo. Western blot analyses carried out using the N terminal-specific-anti Aβ antibody AD54D2, and the C terminal-specific anti-Aβ42 antibodies (clone 295F2) confirmed that the Aβ signal was selectively increased in the hippocampus, but not in the amygdala, in CFC-trained Tg2576 mice. Importantly, the observation that the hippocampal rise of Aβ42 returns to wild-type levels 48 h after the conditioning reveals its transitory nature as well as its suitability to be relaunched by further cognitive activity It is therefore likely that multiple transient episodes of Aβo release triggered every time presymptomatic mice face cognitive tasks can be the starting points of synaptic failure which aggravates over time.

### Neuroinflammation Mediates Structural Plasticity Impairments in Preclinical APPswe/PS1δE9 Mice

Around 4/5 months of age, APPswe/PS1δE9 (δE9) mice exhibit amyloid plaques but do not manifest cognitive alterations and, hence, can be considered as a preclinical model of AD. Using an *in vivo* two-photon microscopy, Zou et al. (2015) imaged spines in the sensory-motor cortex of δE9 mice at rest and found that, consistent with their normal cognitive state, these mice show a large number of intact spines far from the plaques zone. The same authors (Zou et al., 2016) reported however that, at the same age, these mice exhibit a strong deficit in experience-dependent structural plasticity. Specifically, they fail to increase in dendritic spine density following rearing in an enriched environment (EE) and to stabilize these newly formed connections over time. Because amyloid plaques are present at this age point, reduction of BACE1 activity obtained by crossing δE9 with BACE1-KO mice decreased the amyloid burden and restored EE-induced spine formation but did not improve spine stabilization. Differently, the anti-inflammatory drug pioglitazone or the interleukin-1 receptor antagonist IL-1 RA entirely rescued the formation and stabilization of spines suggesting that neuroinflammation phenomena, independent from those triggered by the presence of amyloid plaques, are present in cognitively intact mutants and strongly impair experience-dependent structural plasticity.

## Upstream to Aβo: Role of the Upregulation of Full-Length APP Translation in AD Onset

Close correlations between Aβo release, dysregulation of cytoskeletal proteins like *F*-actin, and disruption in shape, density, and plasticity of spines provide mechanistic interpretations for early synapse destabilization but also give insights into the primary causes of degeneration. In particular, if the formation of Aβo is consequential to pathogenic APP cleavage, the events that trigger the unbalance in favor of amyloid APP processing are likely to contribute to the disease onset. Among those, evidence that upstream to its abnormal proteolysis, full-length APP is overexpressed in familiar (Johnston et al., 1994; Vignini et al., 2013) and sporadic (Matsui et al., 2007) AD patients, and in their respective murine models (Howlett and Richardson, 2009) is getting an increased interest.

This point was early taken by Lanz et al. (2003) who underlined that the developmental effects of APP overexpression on the early loss of spines cannot be ruled out. Interestingly, a longitudinal evaluation of hAPPmRNA and APP levels in the Tg2576 hippocampus revealed that the messenger and the protein are maximally expressed in cognitively asymptomatic 1-month-old mutants (Borreca et al., 2016). This elevation associates with reduced expression of the Fragile-X Mental Retardation Protein (FMRP) and augmented expression of the heteronuclear Ribonucleoprotein C (hnRNP C), two RNA bindings proteins (RBPs) which, in healthy conditions, repress and increase APP translation and expression respectively. The recent observation that polysomal APP mRNA and protein signals associate with decreased levels of the phosphorylated form of initial translation factor eIF2α, a blocker of overall translation, in 1- and 3-month-old Tg2576 mice further indicates that abnormally increased translational mechanisms sustain presymptomatic upregulation of hAPP levels in this model. Confirming that upregulation of translation contributes to AD pathogenesis, pharmacological (salubrinal) restoration of proper translational control in early symptomatic 3-month-old Tg2576 mice reverts structural and functional alterations including spine loss and prevalent LTD at CA1 hippocampal synapses, downregulates their increased levels of the β-secretase enzyme BACE-1, one main determinant of amyloidogenic APP processing, and prevents the manifestation of cognitive alterations (Borreca et al., 2020). Consistent with these findings, compounds like MMP13 (Zhu et al., 2019), which regulates BACE-1 translation, or posiphen, which decreases the production of toxic Aβ by lowering APP translation, are effective in rescuing cognitive deficits in hAPP mutant mice (Lahiri et al., 2007) and sporadic AD patients (Teich et al., 2018). Together, these data suggest that the dysregulation of APP cleavage whose Aβo formation and spines deterioration are the earliest manifestations could largely depend on the abnormal amount of full-length APP to be cleaved at early stages of development.

## Conclusion

The data presented in this short review show that structural and functional degradation of spines mediates the earliest signs of synaptic failure in hAPP mutants and that analyzing the molecular dysfunctions at the origin of spine alterations unveils primary causes of degeneration. Two points in the reported studies need, however, special consideration.

In the AD models examined, synaptic failure is detected as soon as circulating Aβo is present in their brains. Indeed, the causal link between synaptic failure and Aβo is supported by data showing that a majority of molecular dysfunctions which negatively impact dendritic spines in young hAPP mutants (e.g., kalirin-7 disruption) are also observed in wild-type neurons exposed to Aβo alone. Nevertheless, other neurotoxic peptides derived from APP processing like Carboxyl-terminal fragments (Lauritzen et al., 2019), the AICD-amyloid precursor protein intracellular domain (Konietzko, 2012) or the secreted APP ectodomain- sAPPα and (Ishida et al., 1997) which impact synaptic plasticity and are increasingly detected in early AD patients (Perneczky et al., 2013, 2014) could also be involved. Moreover, hAPP mutations in the reported studies were often associated with PS1 mutations suggesting a role for this peptide independent from the facilitation it exerts on APP processing (Thinakaran, 1999).

Of particular relevance among the reviewed data is the observation that cognitively challenged AD mice show synaptic alterations that are undetectable in resting conditions. *In vitro* evidence that neuronal activity increases the formation and release of Aβ peptides in hippocampal neurons overexpressing APP which then shows impairment in excitatory synaptic transmission (Kamenetz et al., 2003) provides a plausible explanatory framework. However, *in vivo* evidence (Pignataro et al., 2019) that cognitive activity enhances Aβ release in the hippocampus of 2-month-old Tg2576 mice and prevents the learning-induced formation of spines in this region indicates that contrary to the beneficial effect of diffuse EE-related sensorial/social stimulation on cognition, focused cognitive activity increases Aβ load in regions supporting high cognitive functions which accelerates the cognitive decline. This aspect should be carefully considered in designing cognitive stimulation protocols aimed at preventing cognitive deterioration at the onset of mild cognitive impairments.

## Author Contributions

The author confirms being the sole contributor of this work and has approved it for publication.

## Conflict of Interest

The author declares that the research was conducted in the absence of any commercial or financial relationships that could be construed as a potential conflict of interest.
